# Comparative Efficacy of Immune Checkpoint Inhibitors and Therapeutic Vaccines in Solid Tumors: A Systematic Review and Meta-Analysis of Randomized Controlled Trials

**DOI:** 10.3390/vaccines13040423

**Published:** 2025-04-17

**Authors:** Rasha Babiker, Adil Farooq Wali, Mohamed El-Tanani, Syed Arman Rabbani, Imran Rangraze, Shakta Mani Satyam, Mohamed Anas Patni, Yahia El-Tanani

**Affiliations:** 1 Department of Physiology, RAK College of Medical Sciences, Ras Al Khaimah Medical and Health Sciences University, Ras Al Khaimah P.O. Box 11172, United Arab Emirates; 2 RAK College of Pharmacy, Ras Al Khaimah Medical and Health Sciences University, Ras Al Khaimah P.O. Box 11172, United Arab Emirates; farooq@rakmhsu.ac.ae (A.F.W.); arman@rakmhsu.ac.ae (S.A.R.); 3 Department of Internal Medicine, RAK College of Medical Sciences, Ras Al Khaimah Medical and Health Sciences University, Ras Al Khaimah P.O. Box 11172, United Arab Emirates; imranrashid@rakmhsu.ac.ae; 4 Department of Pharmacology, RAK College of Medical Sciences, Ras Al Khaimah Medical and Health Sciences University, Ras Al Khaimah P.O. Box 11172, United Arab Emirates; satyam@rakmhsu.ac.ae; 5 Department of Community Medicine, RAK College of Medical Sciences, RAK Medical & Health Sciences University, Ras al Khaimah P.O. Box 11172, United Arab Emirates; mohamedanas@rakmhsu.ac.ae; 6 Royal Cornwall Hospital Trust, NHS, Truro TR1 3LJ, UK; y.el-tanani@nhs.net

**Keywords:** checkpoint inhibitors, therapeutic vaccines, overall survival, solid tumors, immunotherapy, meta analysis, randomized controlled trials

## Abstract

**Background**: Immune checkpoint inhibitors (ICIs) and therapeutic vaccines have emerged as promising immunotherapeutic strategies for solid tumors. However, their comparative efficacy in improving overall survival (OS) remains unclear. This systematic review and meta-analysis aimed to evaluate the efficacy of ICIs and therapeutic vaccines in improving OS in patients with solid tumors. **Methods**: A comprehensive search was conducted across PubMed, Cochrane Library, Embase, and Clinical Trials.gov for randomized controlled trials (RCTs) published between 1 January 2010 and 31 December 2024. Studies comparing ICIs or therapeutic vaccines against control treatments (placebo, standard of care, or active comparators) in adults with solid tumors were included. The primary outcome was OS, and data were pooled using RevMan (web). Risk of bias was assessed using the Cochrane Risk of Bias tool. **Results**: Thirteen RCTs involving 10,991 participants were included. A total of 5722 of them were treated with therapeutic vaccines or checkpoint inhibitors. Therapeutic vaccines demonstrated insignificant improvement in OS, with a pooled mean difference of 1.89 months (95% CI: −0.54–4.31; P = 0.13), although with homogeneity (I^2^ = 0%). ICIs showed a statistically significant OS benefit, with a pooled mean difference of 1.32 months (95% CI: 0.62–2.02; P = 0.0002) and low heterogeneity (I^2^ = 12%). **Conclusions**: Therapeutic vaccines provide a larger but less consistent benefit, whereas ICIs offer modest but more consistent survival advantage. These findings support the need for personalized immunotherapy approaches as well as further research to identify predictive biomarkers and optimize treatment strategies by acquiring deep insights into the TME dynamic and behaviors.

## 1. Introduction

**Figure 1:**
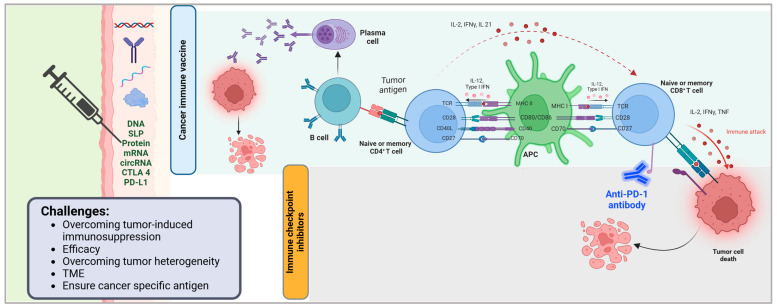
Mechanism of cancer immune vaccines and immune checkpoint inhibitors in tumor immunotherapy. This figure illustrates the immune mechanisms activated by cancer vaccines and immune checkpoint inhibitors (ICIs) in the context of anti-tumor immunity. Cancer vaccines deliver tumor-associated antigens via various platforms (e.g., DNA, synthetic long peptides [SLPs], proteins, mRNA, and circRNA) and immune modulators (e.g., CTLA-4, PD-L1), leading to antigen presentation and priming of B and T lymphocytes. Activated B cells differentiate into plasma cells that produce tumor-specific antibodies, while CD4+ T cells support antigen-presenting cell (APC) activation through co-stimulatory molecules (CD28, CD27, CD40L) and cytokine signaling (IL-2, IFNγ, IL-21). APCs, in turn, stimulate CD8+ T cells through MHC-peptide complexes and co-stimulatory ligands (CD80/CD86, CD70), promoting cytotoxic immune responses. Immune checkpoint inhibitors, such as anti-PD-1 antibodies, block inhibitory signals that suppress T cell activity, thereby restoring T cell-mediated cytotoxicity and enhancing tumor cell killing. Key challenges in optimizing these immunotherapies include overcoming tumor-induced immunosuppression, improving efficacy, addressing tumor heterogeneity, modulating the tumor microenvironment (TME), and ensuring the use of tumor-specific antigens. Created in BioRender.com. Babiker, R. (2025) https://BioRender.com/eyd5k6t.

Cancer vaccines target cancerous cells by stimulating the immune system to recognize and attack tumor specific antigens (Figure 1). These vaccines deliver tumor associated antigens (TAAs) or neoantigens to antigen presenting cells (APCs), such as dendritic cells, which process and present these antigens via MHC molecules to activate CD4+ helper T cells and CD8+ cytotoxic T lymphocytes (CTLs) [1,2,3]. Activated CD8+ CTLs directly kill cancer cells by releasing cytotoxic granules, while CD4+ T cells enhance the immune response by supporting B cell antibody production and amplifying CTL activity [1,2]. For example, the Sipuleucel-T vaccine uses autologous dendritic cells loaded with a prostate-specific antigen to activate T cells against prostate cancer [4]. Some vaccines employ personalized approaches, such as mRNA platforms encoding neoantigens unique to a patient’s tumor to improve specificity [3]. Challenges include overcoming tumor induced immunosuppression and ensuring antigens are cancer specific to avoid attacking healthy cells. Advances in vaccine design, including combination therapies and in situ antigen delivery, aim to enhance efficacy by promoting sustained immune memory and targeting the tumor microenvironment [2,3].

Immunotherapy has greatly changed the treatment of solid tumors by providing more efficient and precise options than chemotherapy and radiation. The most prominent growths in this particular section are immune checkpoint inhibitors (ICIs) and therapeutic cancer vaccines, both of which use bodily immune defense mechanisms to improve the recognition and destruction of cancer cells (Figure 1). These methods have shown great promise in improving the outcomes of patients and changing the strategies in which cancer treatment is approached [5].

Immune checkpoint inhibitors work by focusing on the immune checkpoint proteins that tumors use to bypass immune protection such as programmed cell death protein 1 (PD-1), its ligand (PD-L1), and cytotoxic T-lymphocyte-associated protein 4 (CTLA-4) (Figure 1) [6,7]. ICIs such as nivolumab, pembrolizumab, and ipilimumab block these inhibitory pathways, resulting in ameliorated T cell activity and, therefore, the immune system’s reinvigorated ability to hunt and destroy cancerous cells. Their effectiveness has been clinically studied in a multitude of solid cancers such as melanoma, non-small cell lung cancer, and kidney carcinoma, achieving greater overall survival (OS) and enhanced progression free survival (PFS) than standard treatment options [7,8].

Furthermore, registration progress has been substantial for each product that has been introduced, as they are legal to use in a multitude of interventions, boosting their clinical benefits. At the same time, alongside the innovations in ICIs, cancer therapeutic vaccines surfaced as a plausible immunotherapeutic approach. These vaccines target tumor-associated antigens which can stimulate immune recognition and response against tumors. While therapeutic vaccines have not always been known for their effectiveness, the introduction of personalized neoantigen vaccines and mRNA vaccines certainly sparked interest in the field. Among therapeutic vaccines, sipuleucel-T, a product approved for the treatment of prostate cancer, has shown impressive clinical results. Newer concepts aim at formulation and delivery systems, focusing on better efficacy combined with other types of immunotherapy [8,9].

Combining the effects of therapeutic vaccines with inhibitor monoclonal antibodies has maintained its promise of improving immune responses to solid tumors. One of the predominant problems of cancer immunotherapy is the immunosuppressive TME, an obstacle to immune response and controlling tumor progression [10]. Merging vaccine therapy with ICIs may work by enabling the immune system through vaccination, which is later maintained and boosted by the checkpoint blockade mechanism. This technique in strategy has been demonstrated to improve immune response to the tumor, foster T cell infiltration, and perhaps tear down the resistance barriers that limit the success of monotherapies and anti-ICIs [11].

While some individual trials have been encouraging, there is no broad analysis that directly compares the efficacy of immunotherapy checkpoint inhibitors (ICIs) to therapeutic vaccines. Existing randomized controlled trials (RCTs) have added to the body of knowledge on the usefulness and safety of these procedures, but there is no statement of the effects [12]. In an attempt to close this gap, the current research project intends to perform a systematic review and meta-analysis on the efficacy of ICIs and vaccination for solid tumors. This study has tried to use the available information from RCTs on the treatment of solid tumors to measure survivorship, find heterogeneity, and make recommendations on immunotherapy regulation.

## 2. Materials and Methods

### 2.1. Protocol and Registration

**Figure 2:**
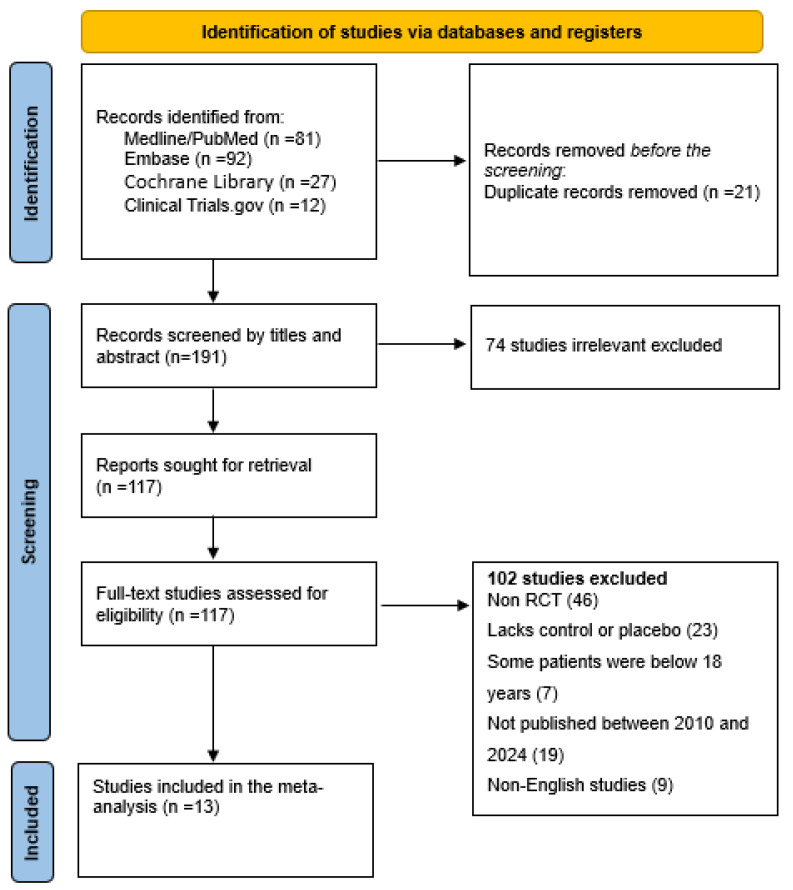
Flow diagram for study identification and inclusion.

The protocol for the systematic review was approved and registered with PROSPERO (CRD42025639024). The Cochrane Hand book for Systematic Reviews of Interventions and the Preferred Reporting Items for Systematic Reviews and Meta-Analyses (PRISMA) were used in this systematic review and meta-analysis (Figure 2).

### 2.2. Eligibility Criteria

As for study identification, all included studies were based on the PICOS criteria (Table S1).

#### 2.2.1. Inclusion Criteria

- Study Design:
  - Only randomized controlled trials (RCTs).
  - Single arm and multi-arm RCTs comparing checkpoint inhibitors or vaccines against a control (placebo, standard of care, or another active treatment).

- Population:
  - Adult males and females (≥18 years) diagnosed with solid tumors (e.g., melanoma, non-small cell lung cancer, renal cell carcinoma, and breast cancer).

- Interventions:
  - Checkpoint inhibitors: Any immune checkpoint inhibitor targeting PD-1, PD-L1, CTLA-4, or other immune checkpoint pathways (e.g., pembrolizumab, nivolumab, ipilimumab, atezolizumab, or durvalumab).
  - Vaccines: Any therapeutic cancer vaccine (e.g., peptide vaccines, dendritic cell vaccines, or mRNA vaccines) used as monotherapy or in combination with other treatments.
  - Studies comparing checkpoint inhibitors versus vaccines, or against a control.

- Comparators:
  - Placebo, standard of care, or another active treatment (e.g., chemotherapy or targeted therapy).

- Outcomes:
  - Primary outcome: overall survival (OS), defined as the length of time from randomization (or start of treatment) until death from any cause. OS was chosen because it is less susceptible to measurement bias and variability in assessment criteria across trials. Studies must report OS or provide sufficient data to calculate OS (e.g., Kaplan–Meier curves, hazard ratios, or survival probabilities).

- Time Frame:
  - RCTs published between 1 January 2010 and 31 December 2024.
  - Ongoing trials with preliminary results available by the end of 2025.

- Language and Publication Status:
  - Only studies published in English.
  - Both peer-reviewed published studies and preprints with available full text data.

- Sample Size:
  - No restriction on sample size, but studies with fewer than 20 participants will be critically evaluated for risk of bias.

#### 2.2.2. Exclusion Criteria

- Non-randomized studies (e.g., observational studies, case reports, and retrospective analyses).
- Studies involving pediatric populations (<18 years).
- Studies focusing on hematologic malignancies or non-solid tumors.
- Studies involving combination therapies where the effect of checkpoint inhibitors or vaccines cannot be isolated.
- Studies without a control group or those comparing non-immunotherapy treatments.
- Abstracts, conference proceedings, and studies without full-text availability unless sufficient data are provided.
- Duplicate publications or secondary analyses of already included trials.

### 2.3. Search Strategy

An extensive systematic search was conducted across multiple electronic databases to identify studies investigating the efficacy of checkpoint inhibitors and therapeutic vaccines in improving overall survival (OS) in patients with solid tumors. Electronic databases, including PubMed, Cochrane Library, Embase, and Clinical Trials.gov, were searched for relevant studies. Reference lists of included studies and key journals in oncology and immunotherapy were also manually searched to ensure comprehensive coverage.

The search is only for English language studies published between 1 January 2010 and 31 December 2024. To ensure the inclusion of the most recent data, the search was repeated beforehand to be sure the most current data are included in the analysis, and other measures to find unpublished studies through clinical trial registries or investigator contacts if needed. Finally, the results are displayed in a PRISMA flow diagram following the PRISMA guidelines and the screening process follows. Finally, a diagram explaining how the number of studies were decided is illustrated at each stage with the following: the number identified, the number screened, the number included, the number excluded, as well as why a study is excluded at each stage (Figure 2).

### 2.4. Data Extraction

Figure 2 is used to elaborate how the identification of eligible studies was performed. During the data extraction process, two independent reviewers are to complete the extraction form. A third reviewer is to go through the data to establish if there are any inconsistency or errors that may arise. The extracted data consists of the study characteristics (i.e., study design), sample size, age, cancer type, intervention, comparators, and overall survival (Table 1).

### 2.5. RoB Assessment

The Cochrane Risk of Bias (RoB) tool was exclusively utilized to evaluate the risk of bias in the included studies for this meta-analysis. This tool is specifically designed for randomized controlled trials (RCTs) and is widely regarded as the gold standard for assessing methodological quality in such studies. The tool systematically evaluates six critical domains: (1) random sequence generation, which assesses the adequacy of the randomization process; (2) allocation concealment, which examines whether the allocation sequence was concealed from participants and investigators; (3) blinding of participants and personnel, which evaluates the effectiveness of blinding procedures to prevent performance bias; (4) blinding of outcome assessment, which ensures that outcome assessors were unaware of the intervention received by participants; (5) incomplete outcome data (attrition bias), which assesses the handling of missing data and its potential impact on results; and (6) selective reporting, which identifies whether all pre-specified outcomes were reported as planned.

The importance of using the Cochrane RoB tool lies in its ability to provide a rigorous and standardized framework for identifying potential biases that could compromise the validity of study findings. By systematically evaluating these domains, the tool ensures that only studies with a low risk of bias are included in the meta-analysis, thereby enhancing the reliability and credibility of the synthesized evidence. This step is critical in minimizing the influence of methodological flaws on the overall results and conclusions, ultimately strengthening the robustness of the meta-analysis and its applicability to evidence-based decision-making.

### 2.6. Statistical Analysis

Data necessary for visualizing the outcomes of the Cochrane Risk of Bias (RoB) assessment was organized and managed systematically on Microsoft Excel. In addition to this, Excel was used to perform numerical calculations, such as computing standard deviations (SDs), for the sake of reliability and consistency in data processing. When data are missing or unclear, the authors of the included studies in such instances were contacted to clarify further and gain the required data. Such cases that were not resolved were explicitly marked, and appropriate imputation methods or sensitivity analyses were carried out to fill such gaps and maintain the integrity of the meta-analysis.

The software platform used for the meta-analysis was RevMan 2024 (web), developed by the Cochrane Collaboration, and widely regarded as adequately reliable. Effect sizes, confidence intervals, and heterogenesis statistics were calculated using RevMan, for example, the I^2^ statistic to quantify the degree of variability across studies. The advantage of this approach is that it fortifies and streamlines a bulletproof synthesis of the data, which allows one to assess the overall weight of the evidence and to identify sources of heterogeneity. By employing these rigorous methodologies, the meta-analysis provided a comprehensive and reliable evaluation of the intervention’s effectiveness, contributing to evidence-based practice and decision-making.

## 3. Results

A systematic search conducted across PubMed, Cochrane Library, Embase, and Clinical Trials.gov identified 212 records (Figure 2). Following a rigorous screening process based on predefined inclusion criteria, 13 high quality studies were selected for inclusion in the final meta-analysis.

**Figure 3:**
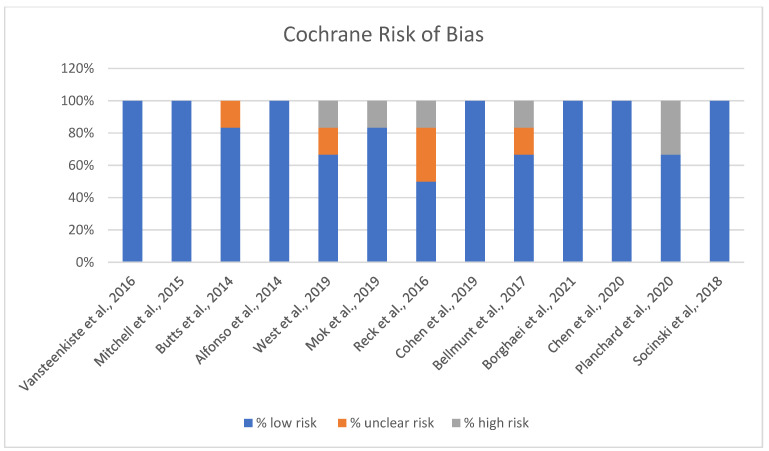
Risk of Bias chart [13,14,15,16,17,18,19,20,21,22,23,24,25].

The Cochrane risk of bias assessment for the studies reveals varied results across different domains of bias (Figure 3).

A significant number of studies show a strong low-risk profile, with 100% of the assessment falling under low risk, suggesting that they generally adhered to robust methodological standards, minimizing bias (Figure 3). A few of the included studies indicated some level of unclear risk, with assessments showing a mix of low and unclear risk percentages. This may suggest potential limitations in certain aspects of the studies, like reporting or performance biases, where more detailed information could be necessary to confirm the risk level. Overall, while the majority of studies demonstrate low risk and adhere to methodological best practices, attention should be paid to the studies with unclear or high-risk ratings to ensure the validity and reliability of the findings.

**Figure 4:**
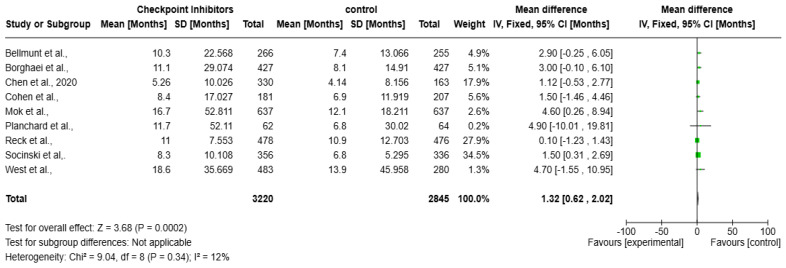
Forest plot of checkpoint inhibitors on improving overall survival in solid tumors [17,18,19,20,21,22,23,24,25].

A meta-analysis over the impact of checkpoint inhibitors on OS in solid tumors patients is presented as the forest plot (Figure 4). Data from several studies used to contribute to the overall effect size, measured in months, are included in the analysis. The overall mean difference in survival is 1.32 months (95% CI: 0.62 to 2.02, *p* = 0.0002), indicating a statistically significant survival benefit. The heterogeneity is low (I^2^ = 12%), suggesting a consistent effect across studies. The weights assigned to individual studies indicate that Socinski et al. (34.5%) contributes the most to the pooled estimate. Most confidence intervals (CIs) show at least a slight survival benefit, except for Planchard et al., where the CI is extremely wide, reflecting uncertainty due to a small sample size.

**Figure 5:**
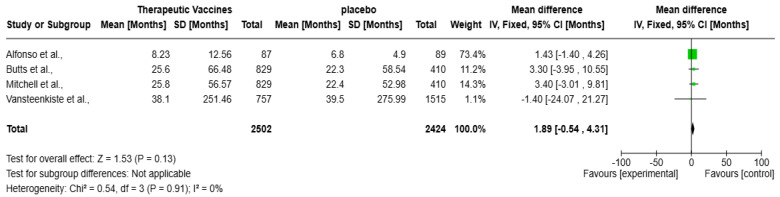
Forest plot of therapeutic vaccines on improving overall survival in solid tumors [13,14,15,16].

The forest plot (Figure 5) presents a meta-analysis evaluating the impact of therapeutic vaccines on OS in patients with solid tumors, encompassing four studies: Alfonso et al., Butts et al., Mitchell et al., and Vansteenkiste et al. Collectively, these studies included a total of 4926 participants. The analysis revealed a statistically non-significant improvement in OS with therapeutic vaccines compared to placebo, with an overall mean difference of 1.89 months (95% CI: −0.54 to 4.31 months; *p* = 0.13). The heterogeneity is negligible (I^2^ = 0%), meaning the studies are highly consistent. Alfonso et al.’s contribution is the largest (73.4%), with no significance, hence, the CI marks zero with an overall mean difference of 1.43 months (95% CI: −1.40 to 4.26 months). Butts et al. and Mitchell et al. show positive but non-significant trends. Notably, Vansteenkiste et al. reported a non-significant mean difference of −1.40 months (95% CI: −24.07 to 21.27 months), presenting a negative effect, adding further uncertainty.

## 4. Discussion

Recently, meta-analyses assessing therapeutic vaccines and checkpoint inhibitors have emerged with terrific progress in dealing with solid tumors, especially relating to the final result of overall survival (OS). They represent two immunotherapeutic strategies in cancer treatment, each with distinct mechanisms. Prospective data from randomized clinical trials are limited, since study protocols have often required that treatment with immune checkpoint inhibitors and immune vaccine to be discontinued if a serious immune related adverse event develops [26].

This result is in line with many studies showing checkpoint inhibitors being a cornerstone in cancer immunotherapy. For example, in the KEYNOTE-024 trial, the PD-1 inhibitor pembrolizumab was found to significantly improve OS compared to chemotherapy in patients with PD-L1 positive NSCLC, with a hazard ratio (HR) of 0.60 [27]. As with CheckMate 067, the median OS was 72.1 months in patients with advanced melanoma receiving nivolumab and ipilimumab, as compared with 36.9 months on ipilimumab alone [28].

Nevertheless, our results showed these positive findings and non-significant low heterogeneity (I^2^ = 12%) in the analysis, which might imply that the benefits of checkpoint inhibitors might be dependent on tumor type or patient populations. For example, Mok et al. [19] developed a large mean difference of 4.60 months for the patients with NSCLC treated with pembrolizumab, whereas Reck et al. [18] found a small mean difference of 0.10 months in a similar population. These discrepancies may be attributed to differences in PD-L1 expression levels, tumor mutational burden, and other biomarkers that influence response to checkpoint inhibition, which aligns with previous findings that PD-1 blockade is particularly effective in tumors with high PD-L1 expression [29]. Similarly, pembrolizumab significantly improved OS in non-small cell lung cancer (NSCLC) patients with PD-L1 expression ≥ 50% (Hazard Ratio (HR): 0.63, *p* < 0.0001) [18].

While immune checkpoint inhibitors (ICIs) can be effective, their benefits are not consistent across all solid tumors. Some research indicates that they show limited effectiveness in “cold” tumors, such as pancreatic or prostate cancer, which do not have enough T cell infiltration. Additionally, immune related adverse events (irAEs) pose a significant concern, necessitating careful selection of patients [30]. To better understand the connection between genetic factors and the risk of these adverse events, larger genome wide association studies may be required. Beyond genetic influences, some studies have explored whether the composition of a patient’s gastrointestinal microbiome is linked to the occurrence of irAEs. Preliminary and emerging clinical evidence suggests that certain bacterial species may correlate with the effectiveness of checkpoint inhibitors, indicating that variations in gut flora that impact host immunity could play a role in the risk of these events [31,32,33].

**Figure 6:**
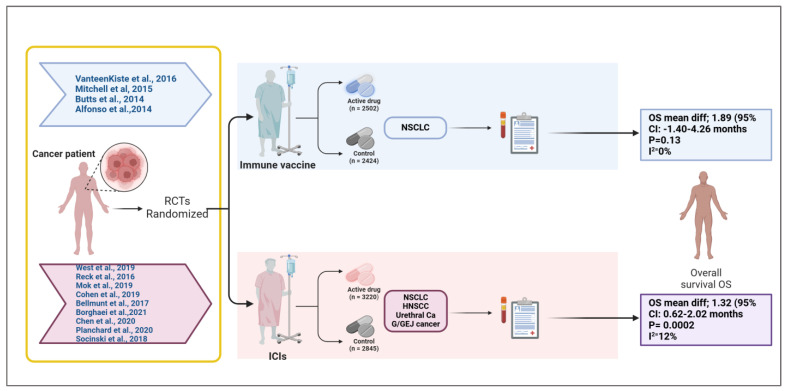
Overview of randomized controlled trials evaluating immune vaccines and immune checkpoint inhibitors in solid tumors [13,14,15,16,17,18,19,20,21,22,23,24,25]. This schematic summarizes the randomized controlled trials (RCTs) included in a meta-analysis assessing the impact of cancer immune vaccines and immune checkpoint inhibitors (ICIs) on overall survival (OS) in solid tumors. The upper panel represents trials investigating immune vaccines in non-small cell lung cancer (NSCLC), with a total of 2502 patients receiving the active vaccine and 2424 in the control arms. The pooled analysis showed a mean OS difference of 1.89 months (95% CI: −1.40 to 4.26; *p* = 0.13; I^2^ = 0%), indicating no statistically significant survival benefit. The lower panel includes trials evaluating ICIs across multiple tumor types (NSCLC, head and neck squamous cell carcinoma [HNSCC], urethral carcinoma, and gastroesophageal junction [G/GEJ] cancer), comprising 3220 patients in the treatment arm and 2845 in the control group. The pooled analysis revealed a statistically significant mean OS improvement of 1.32 months (95% CI: 0.62 to 2.02; *p* = 0.0002; I^2^ = 12%). This figure visually delineates the patient flow, therapeutic categories, cancer types, and associated survival outcomes, emphasizing the differential efficacy profiles of vaccine-based versus checkpoint-based immunotherapies in this study. Created in BioRender.com. Babiker, R. (2025) https://BioRender.com/ehxnobt.

Our meta-analysis evaluated the effect of therapeutic vaccines on improving overall survival in solid tumors; comparing vaccine treatment to a placebo with a mean survival improvement of 1.89 months (95% CI: −0.54 to 4.31, *p* = 0.13) corresponds to the pooled median OS difference observed in RCTs (Figure 5). The analysis examined four studies by Alfonso et al. [16], Butts et al. [15], Mitchell et al. [14], and Vansteenkiste et al. [13], for a total of 4926 participants (experimental arm = 2502 while placebo = 2424), reflect broader trends in vaccine based immunotherapy (Figure 6), for example, the PROSTVAC vaccine in prostate cancer that failed to show OS benefits in a phase III trial (HR: 1.01, *p* = 0.88) [34]. Also, the study of Vansteenkiste et al. targeted Melanoma antigen family A (MAGE-A3), a prototype antigen that is a member of the MAGE-A family of antigens, in lung carcinoma, which did not increase disease free survival compared with placebo [35]. However, the development of an improved MAGE-A DNA immunogen with cross reactivity to multiple family members significantly slowed tumor growth and doubled median mouse survival in a relevant autochthonous melanoma model [35].

A peptide based vaccine, such as Tecemotide, with high sMUC1 and ANA, shows a good possible survival benefit [36] and a potent immunostimulant, enhancing both humoral and cellular immune responses to MAGE-A3 with AS15 [36].

Under certain conditions, non-mutated antigens are capable of giving rise to a therapeutic immune response. On the contrary, neoantigens generated from tumor specific mutations have high immunogenicity with non-central tolerance, but clinical utility is impeded by tumor heterogeneity and the need for personalized vaccine design [37].

Similarly, GVAX vaccines in combination with ICIs in pancreatic cancer have shown modest immunogenicity but no survival advantage in late-stage trials [38]. However, some vaccine strategies show promise in combination therapies.

The CIMAvax-EGF vaccine, which targets the epidermal growth factor, demonstrated prolonged survival in NSCLC when used with chemotherapy [39]. Similarly, dendritic cell (DC) vaccines, such as Sipuleucel-T in prostate cancer, have achieved modest but significant OS improvements (HR: 0.78, *p* = 0.03) [40]. These results indicate that while therapeutic vaccines alone struggle to deliver meaningful survival gains, they may enhance responses in combination regimens. These findings suggest that antigen specific vaccines may have a role in cancer immunotherapy, particularly when combined with other immune modulating agents [26].

Checkpoint inhibitors provide a statistically significant survival benefit, which suggests strong evidence of efficacy, whereas therapeutic vaccines do not, and the confidence intervals for vaccines include both positive and negative values, highlighting uncertainty.

Moreover, checkpoint inhibitors likely benefit a broader range of patients across trials, such as pembrolizumab and nivolumab representing key checkpoint inhibitor immunotherapies used today, but their success remains conditional on tumor type together with PD-L1 expression and other pertinent biomarkers [18,29]. In our study, the survival benefits from therapeutic vaccines slightly exceeded that of checkpoint inhibitors, even though vaccines may have variable effects due to patient specific immune responses.

The mechanism of a cancer vaccine or therapeutic vaccine is to evoke an immune response targeting a specific tumor antigen (cancer cell-expressed protein). This response activates innate immune pathways including Toll-like receptor (TLR) agonists, cytokines, and immune checkpoint inhibitors. But challenges regarding efficacy and how to overcome the tumor heterogeneity, immunosuppressive, tumor microenvironments (TMEs), and immune tolerance are still understudied. The delivery system and type of vaccine might affect the efficiency of vaccine, even as multiple doses sustain an adequate and prolonged immune response [2]. Another factor that limits the impact of vaccine efficacy is the emphasis on specific tumor antigens, which may result in overlooking other relevant antigens that the immune system could target. This oversight can restrict the overall effectiveness in generating a comprehensive antitumor response. Additionally, the genetic diversity of tumors complicates the efficacy of various delivery methods. Approaches such as peptide based, nucleic acid based, protein based, viral vector based, and dendritic cell based vaccines each present unique advantages and challenges that must be taken into account for successful implementation [2].

An important factor to consider when interpreting our findings is the heterogeneity in disease stage and cancer type among patients enrolled in this systematic review. The therapeutic vaccine trials in our analysis targeted patients with varying stages of non-small cell lung cancer (NSCLC), including stage IB, II, and IIIA MAGE-A3-positive tumors, unresectable stage III, and stage IIIb/IV NSCLC. In contrast, trials evaluating immune checkpoint inhibitors (ICIs) included patients with more advanced disease stages, such as stage IV non-squamous NSCLC, extensive stage small cell lung cancer (ED-SCLC), locally advanced or metastatic NSCLC with PD-L1 expression, recurrent or metastatic head and neck squamous cell carcinoma (HNSCC), advanced urothelial cancer, unresectable or recurrent gastric/gastroesophageal junction (G/GEJ) cancer, and wild type metastatic NSCLC (Figure 5).

This variation in tumor stage is clinically relevant, as the tumor burden and tumor microenvironment (TME) differ between early stage and advanced cancers [41].

Patients in the adjuvant or earlier stage setting may have reduced tumor-induced immune suppression, potentially allowing for a more robust response to vaccination. Conversely, those with metastatic or refractory disease often face immune exhaustion, high tumor burden, and immune evasion mechanisms that may blunt the effect of immunotherapy.

These differences may impact both the magnitude and durability of clinical benefit observed with cancer vaccines and checkpoint inhibitors. As the simultaneous assessment of the dynamic shifts of the TME and heterogeneity levels is needed, by incorporating innovative, high level techniques, we can move cancer treatment into the realm of personalized medicine and monitor patients and their response to therapy in real-time clinical settings [42].

Therefore, comparisons across trials should be interpreted with caution, and future studies should consider stratifying results by disease stage to better understand the differential impact of immunotherapy across the cancer spectrum.

Similarly, combination strategies may enhance efficacy, optimizing survival outcomes for specific cancer types. Thus, while both therapies show promise, their comparative effectiveness depends on patient selection and tumor characteristics.

## 5. Limitations and Future Directions

Several concerns exist regarding the observed survival benefits from therapeutic vaccines besides checkpoint inhibitors. Both meta-analyses showed small survival impact from the treatments, since the mean survival improvement reached 1.89 months (see Figure 5) and 1.32 months (see Figure 4). These statistically significant findings demonstrate low clinical significance because patient responses to treatment varied considerably.

Another limitation is the reliance on aggregated data from published studies, which may introduce publication bias. Studies with negative or neutral findings might be underrepresented, leading to an overestimation of treatment benefits. Furthermore, differences in study design, follow-up duration, and endpoints complicate direct comparisons across trials. For example, variations in PD-L1 expression and tumor mutational burden likely influenced outcomes in checkpoint inhibitor studies, highlighting the need for personalized treatment approaches.

Combination therapies, such as pairing checkpoint inhibitors with therapeutic vaccines, chemotherapy, or novel immunotherapies, warrant further investigation to enhance survival benefits. Additionally, real world data and longer follow-up periods will be crucial to assess long-term outcomes and toxicity profiles. Finally, randomized, controlled trials incorporating diverse patient populations are needed to confirm these findings and guide clinical decision-making for immunotherapy in solid tumors.

## 6. Conclusions

The meta-analyses present evidence for the efficacy of therapeutic vaccines and checkpoint inhibitors in improving overall survival in patients with solid tumors. Checkpoint inhibitors and therapeutic vaccines have a clear role in extending survival in clinical practice, but face challenges regarding efficacy; several therapeutic vaccination strategies are under development and are being evaluated clinically.

Future research should focus on optimizing these therapies, identifying predictive biomarkers, and exploring combination strategies to maximize clinical outcomes. These efforts will be critical to realizing the full potential of immunotherapy in oncology and improving survival for patients with solid tumors.

## Figures and Tables

**Table 1:** Study characteristics.

Study ID	Study Design	Sample Size (Exp/Con)	Age	Cancer Type	Interventions	Comparators	Overall Survival (OS)
**Therapeutic vaccines:**							
Vansteenkiste et al., 2016 [13]	Randomized, double blind, placebo controlled trial	2272 patients (1515 in the MAGE-A3 group, 757 in the placebo group	Patients aged at least 18 years	Stage IB, II, and IIIA non-small-cell lung cancer (NSCLC) with MAGE-A3-positive tumors	-MAGE-A3 immunotherapeutic: 13 intramuscular injections of recMAGE-A3 with AS15 immunostimulant -Placebo: Placebo injections	Placebo group	-MAGE-A3 group: 38.1 months (95% CI 27.9–48.4) -Placebo group: 39.5 months (95% CI 27.9–50.4)
Mitchell et al., 2015 [14]	Phase III, randomized controlled trial	1239 patients (Experimental arm n = 829/Control arm n = 410)	Median Age: 61 years, Age Range: 19–89 years	Unresectable Stage III Non-Small-Cell Lung Cancer (NSCLC)	Tecemotide (806 μg lipopeptide)	Placebo (liposome-forming lipids only)	-Experimental: 25.8 mo (95% CI, (23.1–29.4) -Control: 22.4 mo (95% CI, 19.6–25.5) -Hazard Ratio (HR): 0.889 (95% CI: 0.770–1.027).
Butts et al., 2014 [15]	Phase 3, international, randomized, double blind trial	1239 patients (829 to tecemotide, 410 to placebo)	≥18 years	Unresectable stage III non-small cell lung cancer	-Tecemotide (806 μg lipopeptide) injections weekly for 8 weeks, then every 6 weeks until disease progression or withdrawal -Cyclophosphamide (300 mg/m^2^) administered once before tecemotide	Placebo (saline before placebo injections)	-Tecemotide: 25.6 months (95% CI 22.5–29.2) -Placebo: 22.3 months (95% CI 19.6–25.5) -Adjusted HR: 0.88 (95% CI 0.75–1.03), *p* = 0.123
Alfonso et al., 2014 [16]	Randomized, double blind, placebo controlled phase II/III trial	176 patients (87 (Racotumomab-Alum)/89 (Placebo)	≥18 years	Stage IIIb/IV non-small cell lung cancer	Racotumomab-Alum (5 immunizations every 2 weeks, then reimmunizations every 4 weeks)	Placebo	-HR 0.63 (95% CI, 0.46–0.87) Log rank *p* value = 0.004 -Median OS (mo): Racotumomab-alum 8.23 (95% CI, 5.59–10.87); Placebo 6.80 (95% CI, 5.77–7.83)
**Checkpoint inhibitors:**							
West et al., 2019 [17]	Multicenter, randomized, open label, phase 3 study	763 (483 (Atezolizumab + Chemotherapy), 280 (Chemotherapy))	18 years or older	Stage IV non-squamous non-small cell lung cancer	Atezolizumab + Chemotherapy (Carboplatin + Nab-Paclitaxel)	Chemotherapy (Carboplatin + Nab-Paclitaxel)	-Intervention: 18.6 mo (95% CI, 16.0–21.2) -Control: 13.9 mo (95% CI, 12.0–18.7) -Hazard Ratio (HR): 0.79 (95% CI: 0.64–0.98; *p* = 0.033).
Reck et al., 2016 [18]	Phase III randomized controlled trial (RCT), double blind	954 patients (Experimental arm n = 478/Control arm n = 476)	Median age: 62 years (range: 36–85 years).	Extensive-stage small cell lung cancer (ED-SCLC).	-Ipilimumab: 10 mg/kg every 3 weeks for four doses (starting from cycle 3). -Etoposide: 100 mg/m^2^ IV on days 1–3 of each cycle. -Platinum: Cisplatin (75 mg/m^2^) or carboplatin (AUC 5) on day 1 of each cycle.	-Placebo: Administered in the same schedule as ipilimumab, in combination with etoposide and platinum.	-Hazard Ratio (HR): 0.94 (95% CI: 0.81–1.09; *p* = 0.3775). -Median OS: 11.0 months (95% CI: 10.45–11.33) for ipilimumab arm vs. 10.9 months (95% CI: 10.02–11.50) for placebo arm.
Mok et al., 2019 [19]	Randomized, open label, controlled, phase 3 trial	1274 patients (Experimental arm n = 637/Control arm n = 637)	Adults (≥18 years)	Locally advanced or metastatic non-small cell lung cancer (NSCLC) with PD-L1 expression (TPS ≥ 1%)	Pembrolizumab (PD-1 immune checkpoint inhibitor)	Chemotherapy (platinum-based)	-Pembrolizumab: Median OS = 16.7 months (95% CI, 13.9–19.7) -Chemotherapy: Median OS = 12.1 months (95% CI, 11.3–13.3) -Hazard Ratio (HR): 0.81 (95% CI, 0.71–0.93; *p* = 0.0018)
Cohen et al., 2019 [20]	Randomized, open label, phase 3 study	388 patients (181 in pembrolizumab group, 207 in standard of care group)	adults ≥ 18 years implied	Recurrent or metastatic head and neck squamous cell carcinoma (HNSCC)	Pembrolizumab 200 mg every 3 weeks intravenously	Standard of care (methotrexate, docetaxel, or cetuximab intravenously)	Pembrolizumab: Median OS = 8.4 months (95% CI, 6.4–9.4) -Standard of Care: Median OS = 6.9 months (95% CI, 5.9–8.0) -Hazard Ratio (HR): 0.80 (95% CI, 0.65–0.98; nominal *p* = 0.0161)
Bellmunt et al., 2017 [21]	Open label, international, phase 3 trial	521 patients (Experimental arm n = 266/Control arm n = 255)	Adults (≥18 years)	Advanced urothelial cancer (recurrent or progressed after platinum based chemotherapy)	Pembrolizumab (200 mg every 3 weeks)	Chemotherapy (investigator’s choice of paclitaxel, docetaxel, or vinflunine)	-Total Population: -Pembrolizumab: Median OS = 10.3 months (95% CI, 8.0–11.8) -Chemotherapy: Median OS = 7.4 months (95% CI, 6.1–8.3) -Hazard Ratio (HR): 0.73 (95% CI, 0.59–0.91; *p* = 0.002) -PD-L1 CPS ≥ 10% Subgroup: -Pembrolizumab: Median OS = 8.0 months (95% CI, 5.0–12.3) -Chemotherapy: Median OS = 5.2 months (95% CI, 4.0–7.4) -Hazard Ratio (HR): 0.57 (95% CI, 0.37–0.88; *p* = 0.005)
Borghaei et al., 2021 [22]	Randomized, open label, phase III trials	854 patients (427 in nivolumab group, 427 in docetaxel group)	Median age of 61.0 years (range: 37–85) in nivolumab group, 64.0 years (range: 21–85) in docetaxel group	Advanced non- small cell lung cancer (NSCLC), including both squamous and nonsquamous histologies	Nivolumab (3 mg/kg once every 2 weeks)	Docetaxel (75 mg/m^2^ once every 3 weeks)	-Nivolumab Median OS = 11.1 months (95% CI, 9.2–13.1) -Docetaxel: Median OS = 8.1 months (95% CI, 7.2–9.2) -Hazard Ratio (HR): 0.68 (95% CI, 0.59 to 0.78)
Chen et al., 2020 [23]	Randomized, double blind, placebo controlled, phase 3 trial	493 patients (330 in nivolumab group, 163 in placebo group)	Median age of 62 years (interquartile range [IQR]: 54–69) in nivolumab group, 61 years (IQR: 53–68) in placebo group	Unresectable advanced or recurrent gastric/gastroesophageal junction (G/GEJ) cancer	Nivolumab (3 mg/kg every 2 weeks)	Placebo	-Nivolumab Median OS = 5.26 (95% CI, 4.60–6.37) -Placebo: Median OS = 4.14 (95% CI, 3.42–4.86) -Hazard Ratio (HR): 0.62 (95% CI, 0.51–0.76)
Planchard et al., 2020 [24]	Phase 3 RCT	126 patients (62 (Study A), 64 (Study B))	Adults (≥65 years)	metastatic non-small cell lung cancer	Durvalumab (Study A), Durvalumab + Tremelimumab (Study B)	Standard of Care (SoC)	-Durvalumab Median OS = 11.7 (95% CI, 8.2–17.4) -SoC: Median OS = 6.8 (95% CI, 4.9–10.2) -Hazard Ratio (HR): 0.63 ((95% CI, 0.42–0.93)
Socinski et al., 2018 [25]	Randomized trial	692 patients (356 (Atezolizumab + Bevacizumab + Carboplatin + Paclitaxel)/336 (Bevacizumab + Carboplatin + Paclitaxel))	Above 18	Non-Small Cell Lung Cancer (wild type population)	Atezolizumab + Bevacizumab + Carboplatin + Paclitaxel	Bevacizumab + Carboplatin + Paclitaxel	-Intervention: 8.3 mon ((95% CI, 7.7–9.8) -Control: 6.8 mon ((95% CI, 6.0–7.1)

Exp = experimental arm; Con = control or placebo arm.

## Supplementary Materials

The following supporting information can be downloaded at: https://www.mdpi.com/article/10.3390/vaccines13040423/s1, Table S1: Keywords used in the search strategy; Table S2: Search String used in study selection; Table S3: Cochrane risk of bias (For RCTs).

## Abbreviations

The following abbreviations are used in this manuscript:

ICIs	immune checkpoint inhibitors
OS	overall survival
RCTs	randomized controlled trials
PD-1	programmed cell death protein 1
CTLA-4	cytotoxic T-lymphocyte-associated protein 4
MAGE-A	melanoma antigen family A
