# Supplementary material for: Comparative Efficacy of Immune Checkpoint Inhibitors and Therapeutic Vaccines in Solid Tumors: A Systematic Review and Meta-Analysis of Randomized Controlled Trials
Source: Vaccines (Basel). 2025 Apr 17;13(4):423. doi: 10.3390/vaccines13040423 (PMC12030876; doi:10.3390/vaccines13040423)
Supplement: Supplementary file 1 [file vaccines-13-00423-s001.zip › vaccines-3522523-supplementary.pdf]

Supplementary

Table S1. Keywords used in the search strategy.

Category	Keywords	Boolean Operators
Population	"solid tumors," "solid cancers," "carcinoma," "melanoma," "non-small cell lung cancer," "NSCLC," "renal cell carcinoma," "RCC," "breast cancer," "prostate cancer," "colorectal cancer," "gastric cancer"	OR
Interventions	"checkpoint inhibitors," "immune checkpoint inhibitors," "PD-1 inhibitors," "PD-L1 inhibitors," "CTLA-4 inhibitors," "pembrolizumab," "nivolumab," "ipilimumab," "atezolizumab," "durvalumab" "cancer vaccines," "therapeutic vaccines," "peptide vaccines," "dendritic cell vaccines," "mRNA vaccines,"	OR

	"personalized vaccines," "immune vaccines"	
Outcomes	"overall survival," "OS," "survival outcomes," "mortality," "progression-free survival," "PFS," "objective response rate," "ORR," "adverse events," "immune-related adverse events," "irAEs"	OR
Study Design	"randomized controlled trial," "RCT," "randomized clinical trial," "phase III trial," "phase 3 trial," "controlled trial"	OR
Time Frame	"2010–2025," "since 2010," "up to 2025"	AND
Exclusions	"pediatric," "hematologic malignancies," "leukemia," "lymphoma," "myeloma," "non-randomized," "observational study," "case report," "retrospective study"	NOT

Search Algorithms

The following search algorithms was used in databases such as PubMed, Cochrane Library, Embase and Clinical Trials.gov. These algorithms combine the keywords and Boolean operators to maximize sensitivity and specificity.

Table S2. Search String used in study selection.

Electronic Database	Search String Examples
PubMed	("solid tumors" OR "solid cancers" OR "carcinoma" OR "melanoma" OR "non-small cell lung cancer" OR "NSCLC" OR "renal cell carcinoma" OR "RCC" OR "breast cancer" OR "prostate cancer" OR "colorectal cancer" OR "gastric cancer")<br>AND<br>("checkpoint inhibitors" OR "immune checkpoint inhibitors" OR "PD-1 inhibitors" OR "PD-L1 inhibitors" OR "CTLA-4 inhibitors" OR "pembrolizumab" OR "nivolumab" OR "ipilimumab" OR "atezolizumab" OR "durvalumab" OR "cancer vaccines" OR "therapeutic vaccines" OR "peptide vaccines" OR "dendritic cell vaccines" OR "mRNA vaccines" OR "personalized vaccines" OR "immune vaccines")<br>AND<br>("overall survival" OR "OS" OR "survival outcomes" OR "mortality" OR "progression-free survival" OR "PFS" OR "objective response rate" OR "ORR" OR "adverse events" OR "immune-related adverse events" OR "irAEs")<br>AND

	("randomized controlled trial" OR "RCT" OR "randomized clinical trial" OR "phase III trial" OR "phase 3 trial" OR "controlled trial")<br>AND<br>("2010"[Date - Publication] : "2025"[Date - Publication])<br>NOT<br>("pediatric" OR "hematologic malignancies" OR "leukemia" OR "lymphoma" OR "myeloma" OR "non-randomized" OR "observational study" OR "case report" OR "retrospective study")
Embase	("checkpoint inhibitors" OR "immune checkpoint inhibitors" OR "PD-1 inhibitors" OR "PD-L1 inhibitors" OR "CTLA-4 inhibitors" OR "pembrolizumab" OR "nivolumab" OR "ipilimumab" OR "atezolizumab" OR "durvalumab")<br>AND<br>("cancer vaccines" OR "therapeutic vaccines" OR "peptide vaccines" OR "dendritic cell vaccines" OR "mRNA vaccines" OR "personalized vaccines" OR "immune vaccines")<br>AND<br>("solid tumors" OR "solid cancers" OR "carcinoma" OR "melanoma" OR "non-small cell lung cancer" OR "NSCLC" OR "renal cell carcinoma" OR "RCC" OR "breast cancer" OR "prostate cancer" OR "colorectal cancer" OR "gastric cancer")<br>AND

	("overall survival" OR "OS" OR "survival outcomes" OR "mortality" OR "progression-free survival" OR "PFS" OR "objective response rate" OR "ORR" OR "adverse events" OR "immune-related adverse events" OR "irAEs")<br>AND<br>("randomized controlled trial" OR "RCT" OR "randomized clinical trial" OR "phase III trial" OR "phase 3 trial" OR "controlled trial")<br>AND<br>(after:2009 before:2026)
Cochrane Library	("solid tumors" OR "solid cancers" OR "carcinoma" OR "melanoma" OR "non-small cell lung cancer" OR "NSCLC" OR "renal cell carcinoma" OR "RCC" OR "breast cancer" OR "prostate cancer" OR "colorectal cancer" OR "gastric cancer")<br>AND<br>("checkpoint inhibitors" OR "immune checkpoint inhibitors" OR "PD-1 inhibitors" OR "PD-L1 inhibitors" OR "CTLA-4 inhibitors" OR "pembrolizumab" OR "nivolumab" OR "ipilimumab" OR "atezolizumab" OR "durvalumab" OR "cancer vaccines" OR "therapeutic vaccines" OR "peptide vaccines" OR "dendritic cell vaccines" OR "mRNA vaccines" OR "personalized vaccines" OR "immune vaccines")<br>AND<br>("overall survival" OR "OS" OR "survival outcomes" OR "mortality" OR "progression-free survival" OR "PFS" OR "objective response rate" OR

	"ORR" OR "adverse events" OR "immune-related adverse events" OR "irAEs")<br>AND<br>("randomized controlled trial" OR "RCT" OR "randomized clinical trial" OR "phase III trial" OR "phase 3 trial" OR "controlled trial")<br>AND<br>[2010 TO 2025]<br>NOT<br>("pediatric" OR "hematologic malignancies" OR "leukemia" OR "lymphoma" OR "myeloma" OR "non-randomized" OR "observational study" OR "case report" OR "retrospective study")
Clinical trial registry	Condition/disease " solid tumors<br>Other terms "solid cancers" OR "carcinoma" OR "melanoma" OR "non-small cell lung cancer" OR "NSCLC" OR "renal cell carcinoma" OR "RCC" OR "breast cancer" OR "prostate cancer" OR "colorectal cancer" OR "gastric cancer"<br>Intervention/treatment ("checkpoint inhibitors" OR "immune checkpoint inhibitors" OR "PD-1 inhibitors" OR "PD-L1 inhibitors" OR "CTLA-4 inhibitors" OR "pembrolizumab" OR "nivolumab" OR "ipilimumab" OR "atezolizumab" OR "durvalumab" OR "cancer vaccines" OR "therapeutic vaccines" OR "peptide vaccines" OR "dendritic cell vaccines" OR "mRNA vaccines" OR "personalized vaccines" OR "immune vaccines")<br>AND

	("overall survival" OR "OS" OR "survival outcomes" OR "mortality" OR "progression-free survival" OR "PFS" OR "objective response rate" OR "ORR" OR "adverse events" OR "immune-related adverse events" OR "irAEs")

Table S3. Cochrane risk of bias (For RCTs).

Study ID	Random sequence generation	Allocation Concealment	Blinding of Participants	Blinding of Outcome Assessment	Incomplete outcome data (attrition bias)	Selective Reporting
Vansteenkiste et al., 2016	Low risk	Low risk	Low risk	Low risk	Low risk	Low risk
Mitchell et al., 2015	Low Risk	Low Risk	Low Risk	Low Risk	Unclear	Low risk
Butts et al., 2014	Low Risk	Low Risk	Low Risk	Low Risk	Unclear	Low risk
Alfonso et al., 2014	Low Risk	Low Risk	Low Risk	Low Risk	Low risk	Low risk
West et al., 2019	Low Risk	Low Risk	High risk	Unclear	Low risk	Low risk
Mok et al. (2019)	Low Risk	Low Risk	High risk	Low Risk	Low Risk	Low risk
Reck et al., 2016	Low Risk	Unclear	High risk	Unclear	Low Risk	Low risk
Cohen et al., 2019	Low Risk	Low Risk	Low Risk	Low Risk	Low risk	Low risk
Bellmunt et al., 2017	Low Risk	unclear	High Risk	Low Risk	Low risk	Low risk

Borghaei et al., 2021	Low Risk	Low Risk	Low Risk	Low Risk	Low risk	Low risk
Chen et al., 2020	Low Risk	Low Risk	Low Risk	Low Risk	Low risk	Low risk
Planchard et al., 2020	Low Risk	Low Risk	High Risk	High Risk	Low Risk	Low Risk
Socinski et al,. 2018	Low Risk	Low Risk	Low Risk	Low Risk	Low risk	Low risk
